# Identification and validation of a hypoxia-related prognostic and immune microenvironment signature in bladder cancer

**DOI:** 10.1186/s12935-021-01954-4

**Published:** 2021-05-07

**Authors:** Xianchao Sun, Zhen Zhou, Ying Zhang, Jinyou Wang, Xiaofeng Zhao, Liang Jin, Tingshuai Zhai, Xiang Liu, Jiaxin Zhang, Wangli Mei, Bihui Zhang, Ming Luo, Xudong Yao, Lin Ye

**Affiliations:** 1 Department of Urology, Shanghai Tenth People’s Hospital, Tongji University School of Medicine, Shanghai, 200072 China; 2 Department of Urology, The Second Affiliated Hospital of Anhui Medical University, Hefei, 230032 Anhui China; 3 Department of Urology, Shanghai Putuo District People’s Hospital, Tongji University School of Medicine, Shanghai, 200060 China; 4 Department of Urology, Karamay Central Hospital, Karamay, 834000 Xinjiang China

**Keywords:** Bladder cancer, Hypoxia, Prognostic, Immune microenvironment

## Abstract

**Background:**

Bladder cancer is the leading causes of cancer-associated mortality and seriously affects population health. Hypoxia plays a key role in tumor development and immune escape, which contributes to malignant behaviors.

**Methods:**

In this study, we analyzed the RNA-seq and clinical information of bladder cancer patients from The Cancer Genome Atlas (TCGA) database. To investigate the hypoxia-related prognostic and immune microenvironment in bladder cancer, we constructed a hypoxia-related risk model for overall survival (OS). The RNA-seq and clinical data of bladder cancer patients from the Gene Expression Omnibus (GEO) database were used as validation sets.

**Results:**

The hypoxia-related risk signature was significantly correlated with clinical outcomes and could independently predict OS outcomes. Furthermore, the hypoxia-related risk signature could effectively reflected the levels of immune cell type fractions and the expression of critical immune checkpoint genes were higher in the high-risk group compared to the low-risk group. We also validated the expression levels of the prognostic genes in bladder cancer and paracancerous tissue samples through qRT-PCR analysis.

**Conclusion:**

We established a 7 hypoxia-related gene (HRG) signature that can be used as an independent clinical predictor and provided a potential mechanism in bladder cancer immunotherapy.

**Supplementary Information:**

The online version contains supplementary material available at 10.1186/s12935-021-01954-4.

## Background

Hypoxia is a highly crucial process for cells acquiring specific futures to adapt to low oxygen levels [1]. Hypoxia promotes cell proliferation, invasion and regulates immune responses [2, 3]. Globally, bladder cancer is the most common urinary carcinoma, causing more than 199,922 mortalities in 2018 [4]. Despite the current therapeutic options, including surgery, chemotherapy, radiotherapy and some novel immunotherapies, clinical outcomes are still not satisfactory [5]. Bladder cancer treatment is inhibited by its high recurrence rates. Therefore, there is a need to develop precision prediction methods to promote clinical diagnosis and treatment. Hypoxia has been associated with tumor progression and recurrence in bladder cancer [6, 7]. Hypoxic cancer cells regulate tumor microenvironments to facilitate tumor progression and development by releasing exosomes [8]. Circular RNAs involved in adaptive responses to hypoxia, contribute to bladder cancer progression and drug resistance [9]. Recent studies have reported the mechanisms between tumor hypoxia and immune escape, indicating that hypoxia can be used to predict immunotherapeutic outcomes [10–12].

The tumor microenvironment has key roles in bladder cancer. Immune checkpoint molecules are significantly associated with regulation of the tumor microenvironment. Immune checkpoint inhibitors such as programmed cell death protein 1 (PD-1) and programmed cell death-ligand 1 (PD-L1) have been approved for immunotherapeutic management of various cancers. It has been reported that hypoxia induced factor-1 (HIF-1) promotes the expression of PD-L1 by binding the hypoxia response element in the specific proximal promoter. Blockade of PD-L1 under hypoxia enhances myeloid-derived suppressor cells (MDSCs) regulated T cell activation and down-regulates the expression levels of IL-6 and IL-10 [13]. Intermittent hypoxia suppresses autologous T-cell proliferation as well as the cytotoxic activity of CD8^+^ T-cells [14]. Under hypoxia in the tumor environment, cancer cells can adapt to support cellular survival and proliferation. Oxygen-deprived conditions inhibit activation of tumor-infiltrating lymphocytes, leading to an immunosuppressive environment and tumor immune escape. Cancer cells are able to maintain their metabolism under hypoxic conditions and metabolically outperform tumor-infiltrating T cells for glucose, resulting in cancer progression and suppression of T cell activity [15]. Studies have evaluated the therapeutic pathways for blocking hypoxia-associated transcription factors. Specifically, some strategies targeting HIF-1ɑ factors have been implicated in cancer biology and enhancement of immunotherapeutic sensitivity [16]. Therefore, tumor hypoxia is a potential therapeutic target in immunotherapy.

In this study, we developed a hypoxia-related risk signature as a prognostic symbol to reflect the immune landscape in bladder cancer. We screened 7 HRGs from The Cancer Genome Atlas bladder cancer cohort (TCGA-BLCA) that were significantly correlated with OS outcomes of bladder cancer. Samples were divided into high-risk and low-risk groups according to the median risk score. Furthermore, survival and Cox analyses were performed to estimate the prognostic value of the hypoxia risk model. The different mechanisms involved in signaling pathways and the fractions of immune cell types between the high-risk and low-risk groups were also evaluated. This study aimed at analyzing the expression levels of HRGs in bladder cancer and establishing their potential prognostic values. Importantly, we constructed and verified a hypoxia-related signature that could improve the precise prognosis prediction in bladder cancer.

## Materials and methods

### Data source

The RNA-seq transcriptome data and clinical characteristics of the BLCA cohort were downloaded from the TCGA (https://portal.gdc.cancer.gov/) database. Gene expression profiles and clinical data in GSE32894 were obtained from the GEO (https://www.ncbi.nlm.nih.gov/geo/) database. Detailed patient data are presented in Additional file 7: Tables S1 and Additional file 8: Table S2.

### Construction a protein–protein interaction (PPI) network

The PPI network was established using the STRING database (http://string-db.org). The cytoscape software (https://cytoscape.org/) platform was used to visualize and integrate these associated protein networks. The Network Analyzer plug-in was used to calculate the node degree between these networks and define the key genes in the network.

### Development and validation the hypoxia-related prognostic signature

Prognostic HRGs in bladder cancer obtained from univariable and multivariable Cox regression were screened to calculate the each patient’s risk score. Risk scores were calculated as:

$$Risk \, Score \, = \, Expression_{gene1} \times \, Coefficient_{gene1} + \, Expression_{gene2} \times \, Coefficient_{gene2} + \, ... \, Expression_{genen} \times \, Coefficient_{genen}$$

Next, we investigated whether the risk score was correlated with patients’ OS outcomes.

### Evaluation of immune cell type fractions

CIBERSORT (https://cibersort.stanford.edu/) was used to evaluate proportions of tumor-infiltrating lymphocytes in a mixed cell population. A total of 22 immune cell types in this tool were used to estimate relative abundance of immune cell infiltration between low- and high-risk groups.

### Gene set enrichment analysis (GSEA)

Based on the median value of risk scores, patients were divided into low-risk and high-risk groups. GSEA was performed to determine the different pathway signaling genes between the groups. Analysis was performed by GSEA3.0 (http://www.broad.mit.edu/gsea/) and nominal p < 0.05 was considered statistically significant.

### Clinical patients and bladder specimens

A total of 45 paired cancer and paracancerous tissues samples were obtained from bladder cancer patients subjected to radical cystectomy at the Shanghai Tenth People’s Hospital (Shanghai, China). Patients were diagnosed according to WHO classifications without any preoperative treatment. Tissue specimens were evaluated by at least two experienced pathologists. Tissues were immediately frozen in liquid nitrogen and stored at − 80 °C. Clinical information and pathological features of bladder cancer samples are shown in Additional file 9: Table S3. All patients provided an informed consent before inclusion in the study, and ethical approval was obtained from the Ethics Committee of Shanghai Tenth People’s Hospital. Total RNA was extracted from samples and qRT-PCR analysis performed to validate the expression levels of the 7 HRGs in human samples.

### RNA extraction and quantitative real-time (qRT-PCR)

Total RNA was extracted using the Trizol reagent (Invitrogen, CA, USA) according to the manufacturer’s instructions. Next, qRT-PCR was performed using SYBR-Green Mix (Vazyme, Nanjing, China) with different primers (Sangon Biotech, China). Glyceraldehyde-3-phosphate dehydrogenase (GAPDH) was used as an internal standard. Fold-changes were calculated by the 2^−ΔΔCt^ method. Primer information is shown in Additional file 10: Table S4.

### Immunohistochemistry (IHC)

Cancer and paracancerous tissue samples were fixed in formalin, embedded in paraffin and cut into 4 μm slices. After dewaxing, rehydration, and antigen retrieval, the slices were incubated with specific primary antibodies against EGFR and SLC2A3 (Proteintech, USA). Images were obtained using a microscope (Nikon, Japan).

### Statistical analysis

Data analyses were performed using R programming language (https://www.r-project.org/). Univariate and multivariate Cox proportional hazard regression analyses were used to screen genes and evaluate the correlation between risk scores and OS. Receiver operating characteristic (ROC) analysis was performed using the “survival_ROC” package to assess the sensitivity and specificity of the risk model to predict survival outcomes. The area under the ROC curve (AUC) was used to show prognostic accuracy. A Student’s t test was used to compare differences between different risk groups. p ≤ 0.05 was considered statistically significant.

## Results

### Establishment of the hypoxia-related risk signature to predict bladder cancer prognosis

**Fig. 1:**
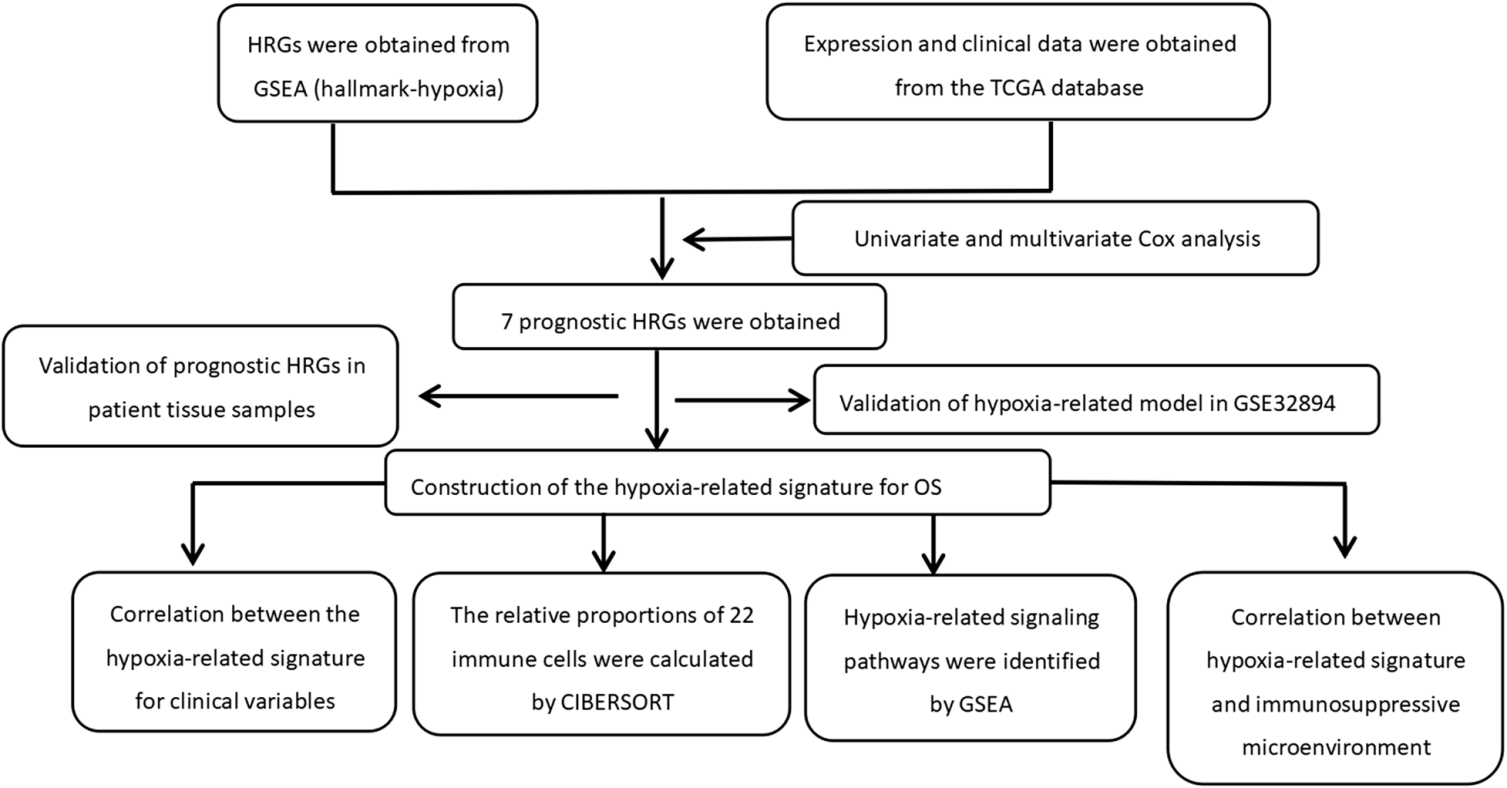
The flowchart of this study

**Fig. 2:**
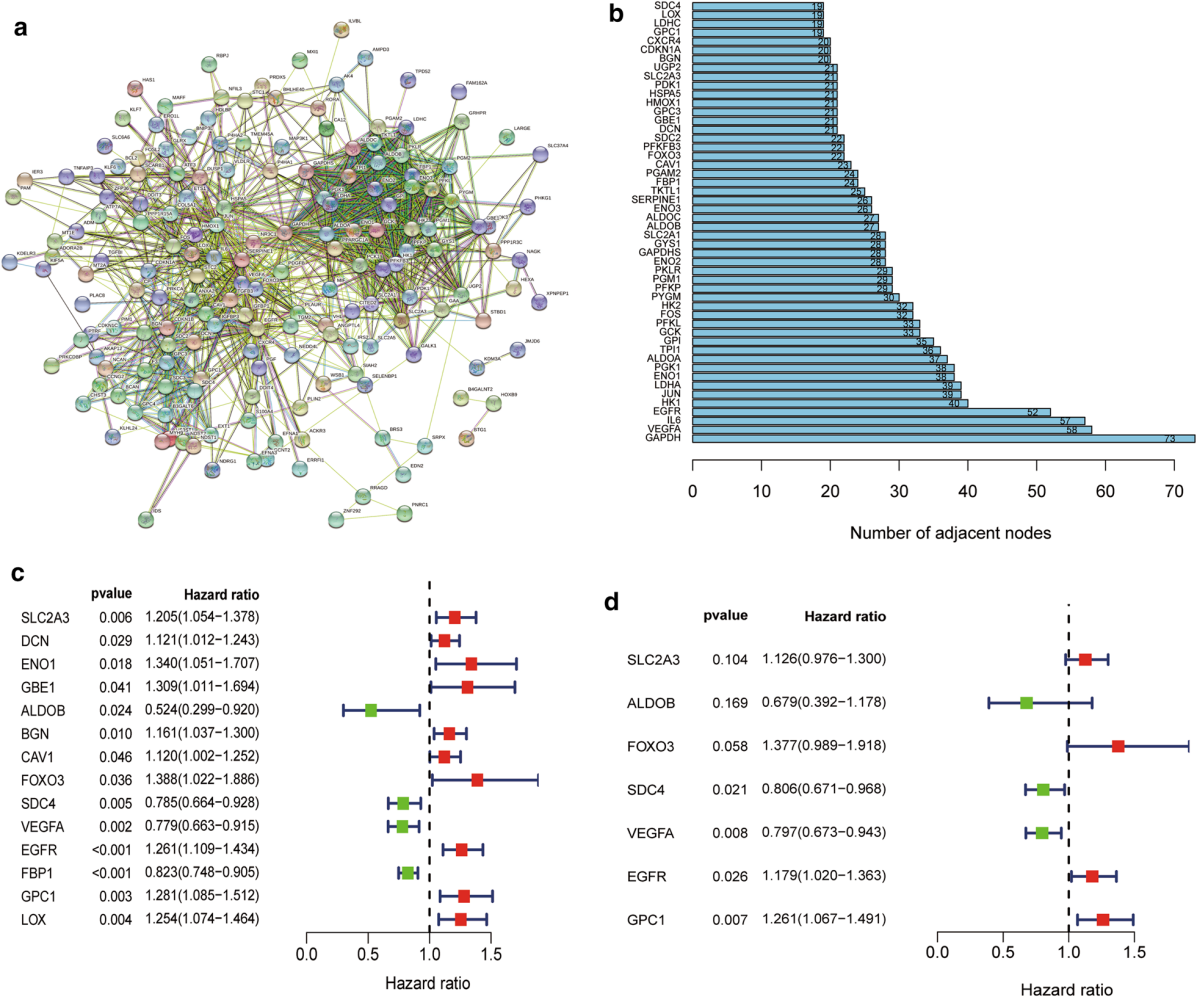
Identification of hypoxia-related genes in bladder cancer. **a** Protein–Protein network interactions including 200 hypoxia-associated genes. **b** The 50 genes with the most associated interaction degrees were selected. **c** Univariate Cox regression analysis of hypoxia-related genes. **d** Multivariate Cox regression analysis of hypoxia-related genes

Figure 1 shows the flow chart of this study. The RNA-seq and clinical prognostic information data were downloaded from the TCGA-BLCA cohort. The hypoxia-related gene set was obtained from GSEA (hallmark-hypoxia). Then, we performed PPI network analysis using the STRING online database and Cytoscape software to further establish the interactions between these genes (Fig. 2a). A total of 50 genes with the most significant interaction degrees were screened (Fig. 2b).

To construct the hypoxia-related risk model, univariate and multivariate Cox analyses were performed using the top 50 genes in the TCGA-BLCA cohort. In the univariate Cox analysis, 14 HRGs were significantly associated with patients’ OS outcomes (Fig. 2c). After multivariate Cox regression analysis, 7 HRGs were identified and selected to construct the OS signature (Fig. 2d). The risk score signature was developed as: risk score = (0.119 × SLC2A3 expression level) + (− 0.387 × ALDOB expression level) + (0.320 × FOXO3 expression level) + (− 0.216 × SDC4 expression level) + (− 0.227 × VEGFA expression level) + (0.165 × EGFR expression level) + (0.232 × GPC1 expression level).

### Prognostic significance of the hypoxia-related risk signature in bladder cancer patients

**Fig. 3:**
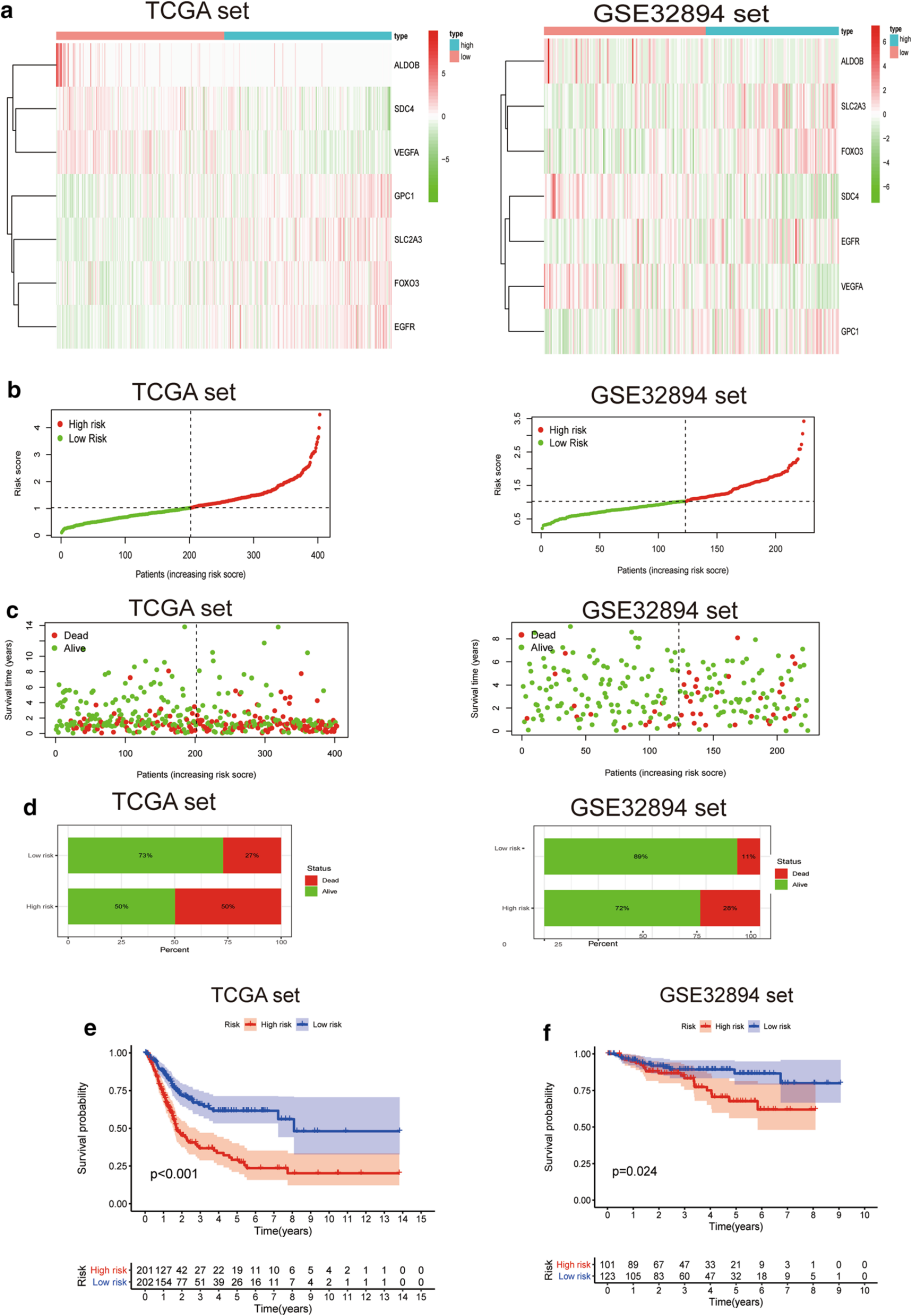
Prognostic significance of the hypoxia-related risk signature in bladder cancer. **a** A heatmap for the expression levels of the 7 hypoxia genes between low- and high-risk groups in the TCGA and GSE32894 datasets. **b** Distribution of the risk scores of bladder cancer patients. **c** Patient status distribution in the low- and high-risk groups. The dot represents patient status as ranked by the increasing risk score. The *X* axis is patient number while the *Y* axis is survival time. **d** Mortality rates of the low- and high- risk groups. **e**, **f** The prognostic significance of the hypoxia signature in TCGA and GSE32894 databases

Figure 3a shows the expression levels of the 7 HRGs in low- and high-risk groups. Risk scores of patients in the low- and high- risk groups were visualized (Fig. 3b). As the hypoxia risk score increased, the mortality rate for bladder cancer patients increased (Fig. 3c, d). Moreover, KaplanMeier analysis was used to evaluate the prognostic significance of the hypoxia-related risk signature. A high hypoxia risk score was correlated with poor OS outcomes in the TCGA and GSE32894 sets when compared to the low risk score (Fig. 3e, f).

### The hypoxia-related risk signature for OS is an independent prognostic value for bladder cancer patients

**Fig. 4:**
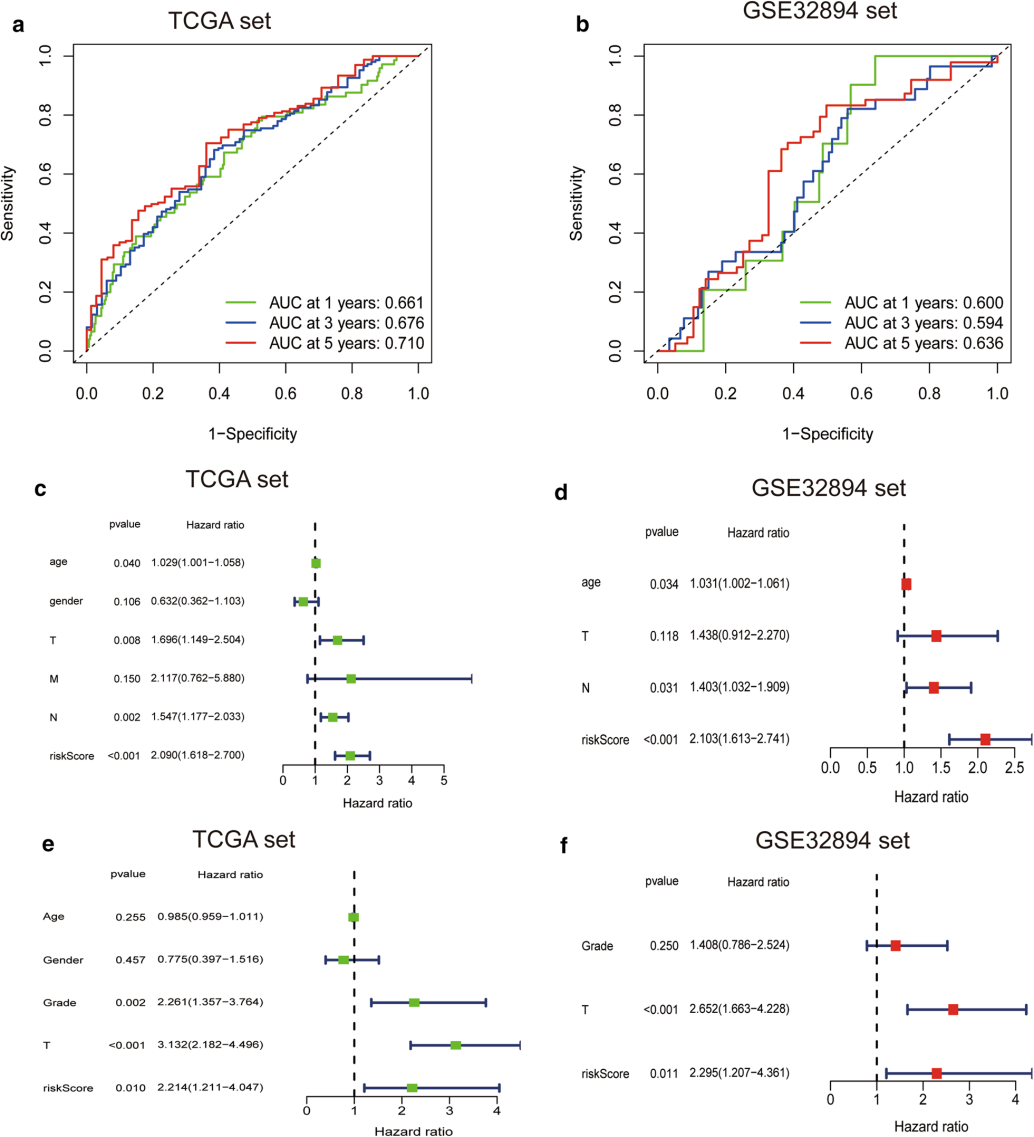
The hypoxia-related signature for OS is an independent prognostic factor for bladder cancer. **a**, **b** ROC curves showing the predictive efficiency of the hypoxia-related risk signature on the 1-, 3-, and 5-years survival rate. **c**–**f** Univariate and multivariate Cox analysis of correlations between the risk score for OS and clinical variables

To determine the predictive accuracy of the hypoxia-related risk signature, we used the ROC curve to assess the model. The AUC of the signature for the prediction of 1-, 3-, and 5-year OS were 0.661, 0.676 and 0.710, respectively, in the TCGA set and 0.600, 0.594, 0.636, respectively, in the GSE32894 set (Fig. 4a, b). Moreover, we compared the 5-year ROC curve with other clinical variables (Additional file 1: Figure S1). Then, univariate and multivariate Cox analyses were used to determine the independent prognostic value of the hypoxia-related risk signature for OS. Univariate Cox analyses revealed that the risk score was associated with OS and other variables including age, gender and WHO grade (Fig. 4c). Multivariate Cox analysis revealed that the risk score was independently correlated with OS (Fig. 4e). These results were validated using the GSE32894 set (Fig. 4d, f).

### Relationships between the prognostic signature with clinicopathological variables

**Fig. 5:**
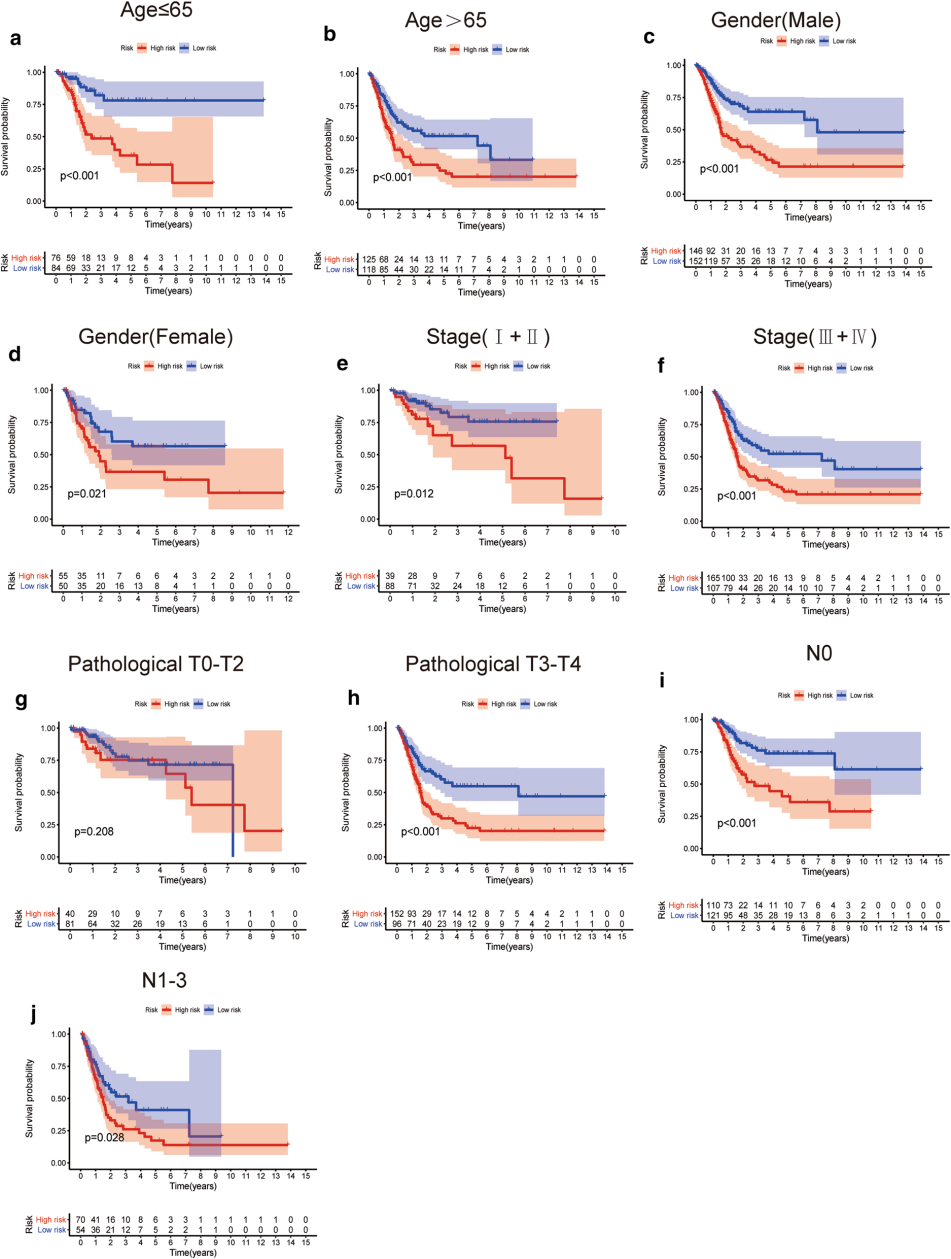
Kaplan–Meier survival curves for the low- and high-risk groups stratified by clinicopathological variables in the TCGA dataset. **a**, **b** Age. **c**, **d** Gender. **e**, **f** Stage. **g**, **h** Pathological T stage. **i**, **j** N Stage

To determine whether the prognostic signature was correlated with clinicopathological variables in TCGA-BLCA. Bladder cancer patients were stratified according to age, gender, stage, T stage and N stage. High-risk patients with those clinical parameters exhibited significantly shorter OS outcomes than low-risk patients (Fig. 5), suggesting that the hypoxia-related signature could be applicable to clinical factors. The correlation between the clinicopathological variables and the prognostic signature in GSE32894 is shown in Additional file 2: Figure S2.

### Correlation between the hypoxia-related risk signature and immune cell infiltration

**Fig. 6:**
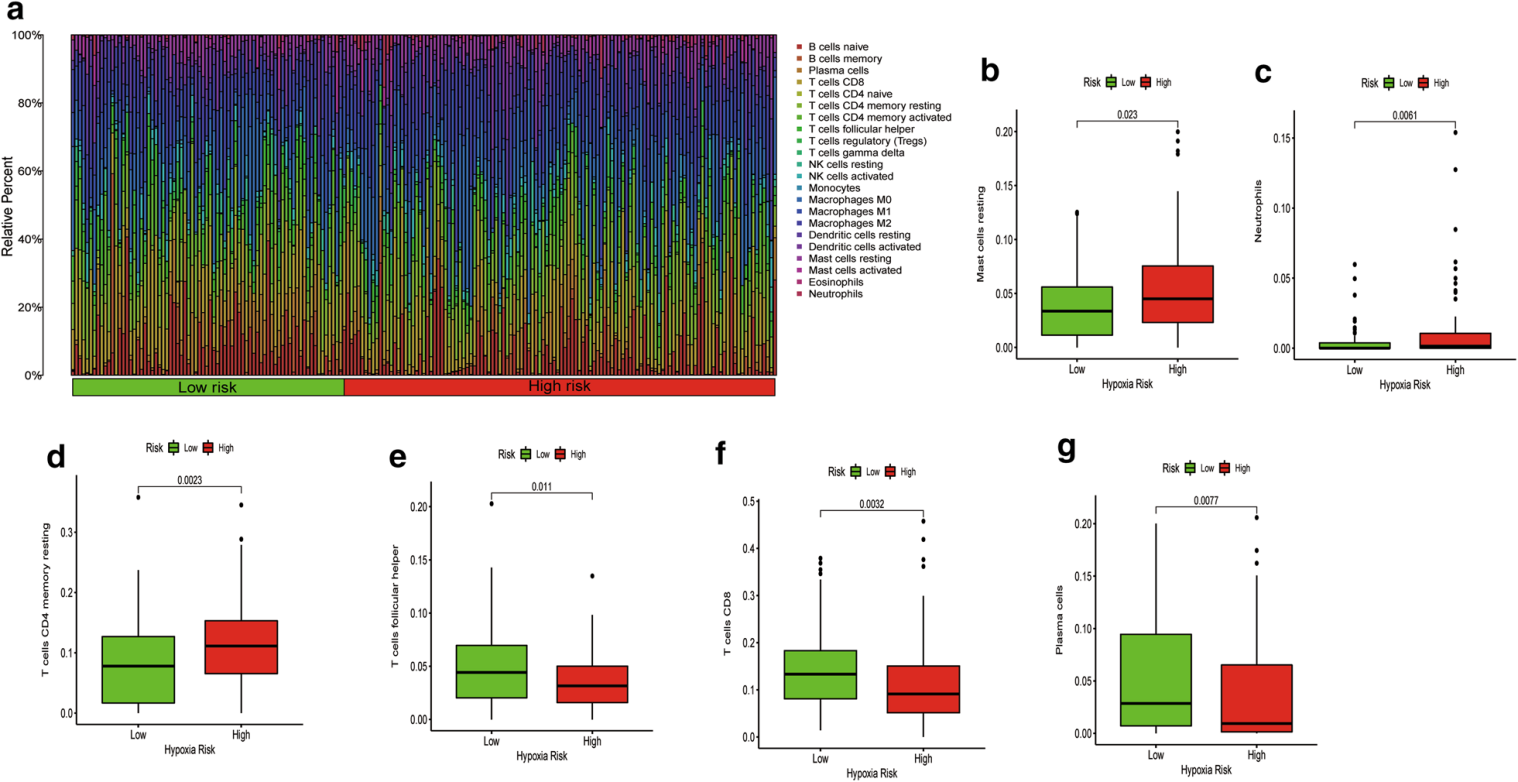
Immune landscape between low and high hypoxia risk bladder cancer patients. **a** The proportion of immune infiltration levels in low and high hypoxia risk patients. **b**–**g** Box plots showing significantly different immune cells between low and high hypoxia risk patients

To investigate the utility of the risk signature in reflecting the immune cell environment. We used CIBERSORT analysis to estimate expression levels of the 22 immune cell types infiltration between different risk levels of bladder cancer patients. Immune cell population among the patients is shown in Fig. 6a. The low hypoxia risk patients exhibited higher levels of proportions of follicular helper T cells (p = 0.011), CD8 T cells (p = 0.0032), and plasma cells (p = 0.0077) when compared to the high-risk group (Fig. 6e–g). However, a higher proportion of mast resting cells (p = 0.023), neutrophils (p = 0.0061), and CD4 memory resting T cells (p = 0.0023) were enriched in the high-risk group (Fig. 6b–d). Correlations between the hypoxia-related risk signature and immune cell infiltration are shown in Additional file 3: Figure S3. Detailed correlations of the 22 immune cells and the hypoxia-related signature are presented in Additional file 11: Table S5.

### Functional analysis of the prognostic signature

**Fig. 7:**
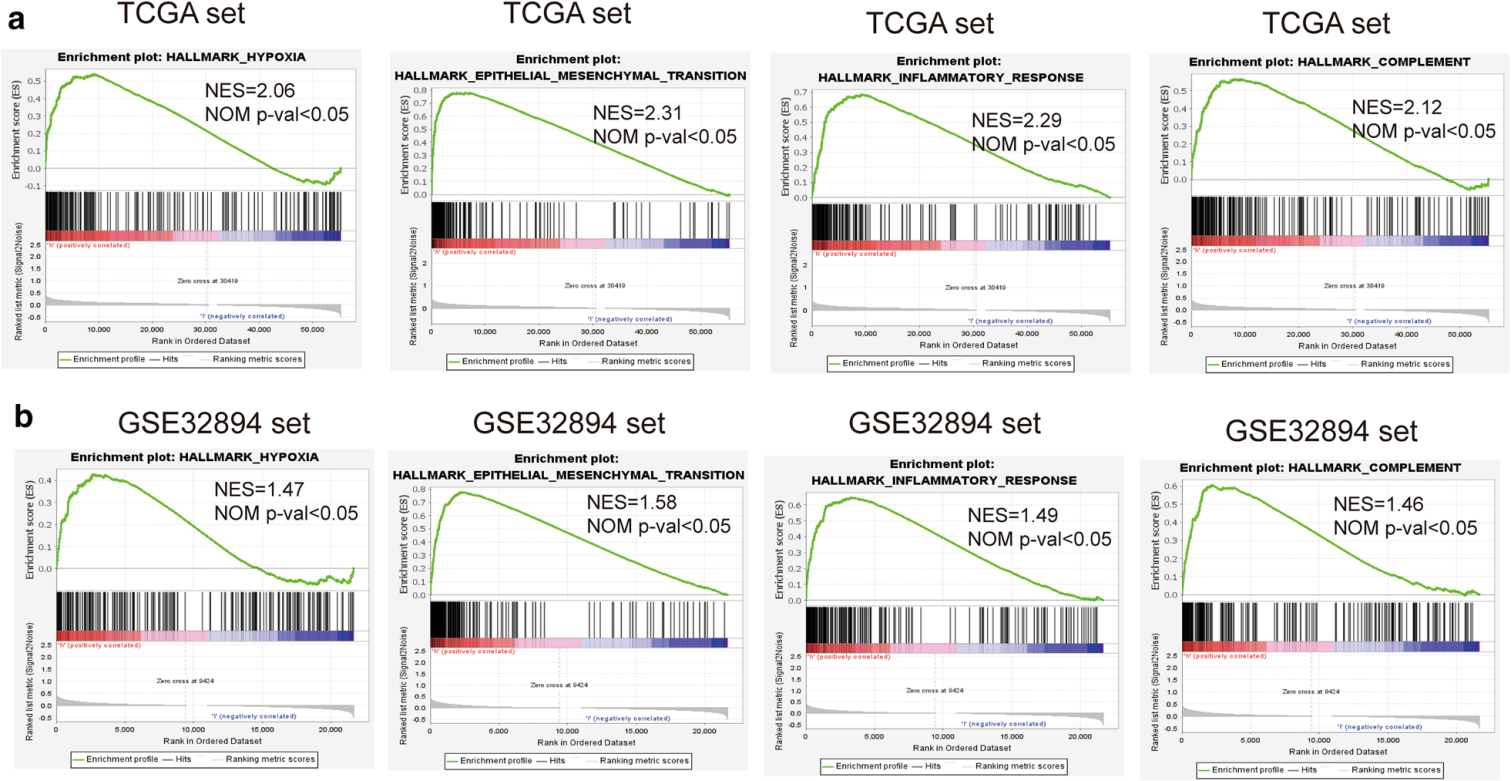
GSEA comparisons of the low and high hypoxia risk groups. Common functional gene sets enriched in the high risk group compared to low risk group in TCGA (**a**) and GSE32894 sets (**b**)

We further verified the underlying mechanisms involved in the low- and high-risk groups. GSEA analysis revealed that signaling pathways such as hypoxia, epithelial-mesenchymal transition, inflammatory responses and complement were significantly enriched in the high risk group (Fig. 7).

### Associations between the hypoxia-related risk signature and the immunosuppressive microenvironment

**Fig. 8:**
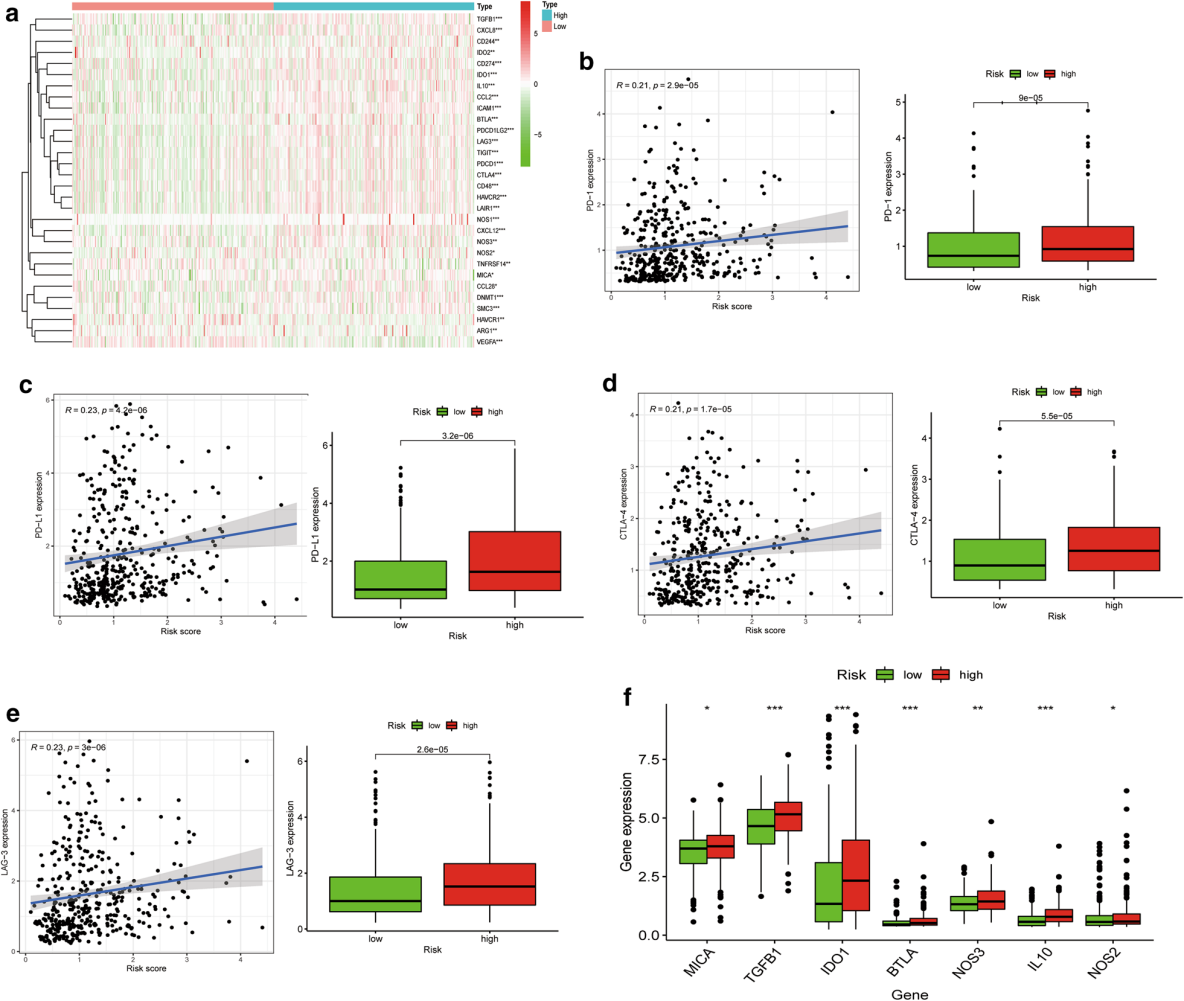
The hypoxia risk score was associated with the immune microenvironment. **a** Heatmap of related negative genes involved in the regulation of the cancer-immunity cycle in low and high risk groups in TCGA-BLCA. **b** Correlation between PD-1 expression and hypoxia risk score. **c** Correlation between PD-L1 expression and hypoxia risk score. **d** Correlation between CTLA-4 expression and hypoxia risk score. **e** Correlation between LAG-3 expression and hypoxia risk score. **f** Tumor immunosuppressive cytokine expression in low and high hypoxia risk groups. *p < 0.05, **p < 0.01, and ***p < 0.001

Immunotherapy is a promising option for advanced urothelial carcinoma. The cancer-immunity cycle regulates cancer cells and immune responses, which affects utility to immune therapies. The cancer-immunity cycle process is initiated by the release of cancer-associated antigens. Then, associated antigens are identified by dendritic cells and transferred to lymph nodes, accompanied by T cell activation. Effector cells then migrate and infiltrate the tumor stroma, where they specifically recognize and eliminate cancer cells. Every step of the cycle needs the coordination of stimulatory and inhibitory factors. In this study, we focused on the genes that negatively mediate the process in low- and high- risk groups. Related gene signatures were obtained from the Tracking Tumor Immunophenotype website (http://biocc.hrbmu.edu.cn/TIP/index.jsp). Genes enriched in negative regulation of the cycle were mostly elevated in the high risk score group (Fig. 8a), indicating that high hypoxia risk patients are associated with poor immunotherapeutic efficacies.

Moreover, the association between the hypoxia-related signature and expression levels of important immune checkpoint genes (i.e., PD-L1, PD-1, CTLA-4 and LAG-3) were evaluated. As shown in Fig. 8b–e, the four immune checkpoints were correlated with the hypoxia risk score and upregulated in the high hypoxia risk group. Furthermore, we evaluated the expression levels of some immunosuppressive genes in the low- and high- risk groups. The high hypoxia risk group exhibited significantly elevated mRNA expression levels of immunosuppressive genes (Fig. 8f).

These results reveal that high hypoxia risk patients may develop an immunosuppressive microenvironment and become insensitive to immunotherapy.

### Validation of the 7 HRGs expression in bladder cancer tissue samples

**Fig. 9:**
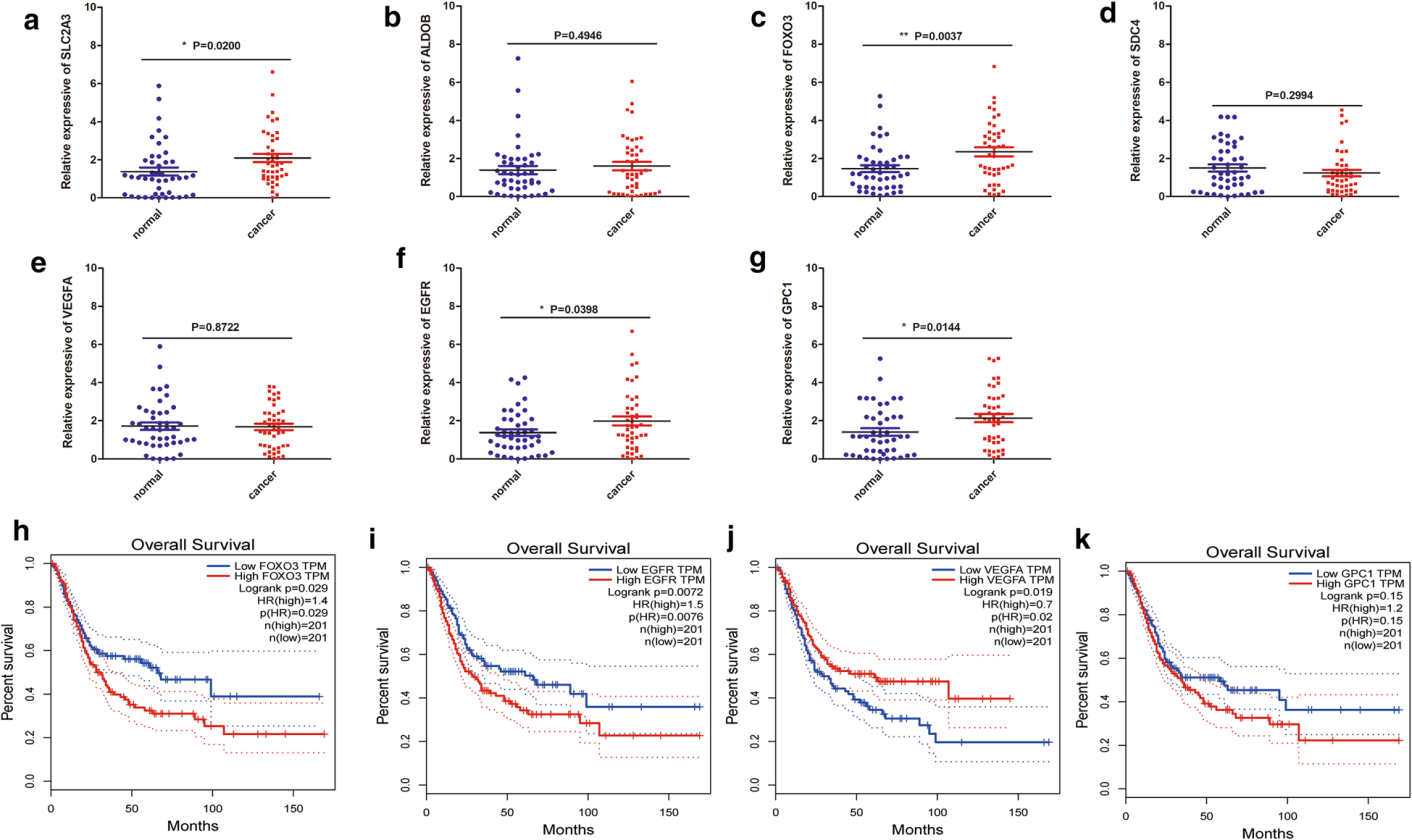
Validation of gene expressions using qRT-PCR. **a**–**g** qRT-PCR analysis validation of the expressions of SLC2A3, ALDOB, FOXO3, SDC4, VEGF, EGFR and GPC1 in paracancerous and cancer tissues. **h**–**k** GEPIA Survival analysis of FOXO3, EGFR, VEGFA and GPC1. *p < 0.05, **p < 0.01

Figure 9a–g shows that expression levels of SLC2A3, FOXO3, EGFR and GPC1 were elevated in tumor samples. There was no significant difference in the expression levels of ALDOB, SDC4 and VEGFA. Moreover, the Gene Expression Profiling Interactive Analysis (GEPIA) database was used to analyze the correlations between the HRGs and OS outcomes in TCGA-BLCA. High expression levels of FOXO3, EGFR, and GPC1 as well as low expressions of VEGFA were closely correlated with poorer survival outcomes of bladder cancer patients (Fig. 9h–k). Expression levels of the other genes did not exhibit significant differences in survival outcomes between the two risk groups (Additional file 4: Figure S4). Immunohistochemistry data from the Human Protein Atlas were used to verify the expression of HRGs in normal and tumor tissues (Additional file 5: Figure S5). Moreover, we also performed IHC analysis to detect the expression levels of EGFR and SLC2A3 and the result showed that the expression of the two genes were higher in cancer tissues compared to paracancerous tissues (Additional file 6: Figure S6)

## Discussion

Globally, bladder cancer is the 10th most common cancer and is associated with high morbidity and mortality rates. Due to tumor recurrence and drug resistance, clinical outcomes have not exhibited an improvement within the past few years [17]. Precise diagnostic and predictive methods are urgently needed to promote the treatment and prognosis of bladder cancer. Immunotherapy is a novel treatment method for bladder cancer, which acts by blocking immune checkpoints, however, its therapeutic efficacies are not very high [18].

Tumor hypoxia promotes the growth of tumor cells and is involved in the conversion to the malignant phenotype. In addition, tumor hypoxia is a prognostic factor for cancer. Lin et al. constructed a prognostic model of HRGs in glioma [19]. Zhang et al. defined a hypoxia-related signature in pan-cancer using multi-omics data [20]. We established a hypoxia-related risk signature and evaluated its prognostic value in predicting OS. Moreover, we evaluated the immune microenvironment among the low and high risk score groups. We used univariate Cox regression to determine the HRGs that were correlated with bladder cancer prognosis. A total of 14 HRGs were found to be significantly correlated with prognosis. Multivariate Cox regression analysis identified 7 HRGs (SLC2A3, ALDOB, FOXO3, SDC4, VEGF, EGFR, GPC1), which were used to construct the risk score model. High expression of solute carrier family 2 facilitated glucose transporter member 3 (SLC2A3) is closely associated with poor prognosis of papillary thyroid cancer [21] and colorectal cancer [22]. ALDOB has been found to be upregulated in liver metastases and it enhances fructose metabolism [23]. FOXO3 promotes tumor angiogenesis in neuroblastoma [24]. SDC4 gene silencing attenuates the epithelial-mesenchymal transition and promotes apoptosis of papillary thyroid cancer cells [25]. VEGF is primarily responsible for angiogenesis and it promotes cell proliferation [26]. EGFR can be used as a potential therapeutic target for muscle-invasive bladder cancer presenting a basal-like phenotype [27]. GPC1 is an independent prognostic factor for oesophageal squamous cell carcinoma [28].

GSEA showed that genes involved in the regulation of hypoxia, epithelial-mesenchymal transition and inflammatory responses were more enriched in the high-risk group compared to the low-risk group. However, the mechanisms by which these genes are negatively expressed in the low-risk group should be further evaluated. In addition, we found that the hypoxia-related risk signature can independently predict the prognosis of bladder cancer patients. High risk score patients were associated with worse outcomes. We validated the applicability of the hypoxia-related risk signature in the GSE32894 dataset. Furthermore, qRT-PCR analysis revealed that SLC2A3, FOXO3, EGFR and GPC1 were upregulated in tumor samples.

Hypoxia can affect the functions of immune cell types, thereby directly or indirectly inducing tumour development. Under hypoxic environments, macrophages synthesize chemokines and cancer cells to attract regulatory T-cells from circulation and suppress anti-tumour responses by other T-cells [29]. Hypoxic microenvironments suppress anti-tumor immune effector cells and facilitate immune escape for the growth of tumor cells [30]. Hypoxia promotes FOXP3 transcription factor expression levels, which is a potent regulator of Treg cells [31]. Moreover, hypoxia enhances the expression of CCL28 and TGF-β, which are involved in chemo-attracting Treg cells, regulating the inhibition efficacy of Teff cell responses, as well as contributing to angiogenesis and tumor tolerance [32]. Tumor-associated macrophages can promote malignant progression by inducing angiogenesis in the tumor hypoxic environment. Hypoxia was shown to significantly promote the positive percentage of PD-L1 related myeloid-derived suppressor cells (MDSCs) in tumor-bearing mice [13]. In this study, CIBERSORT revealed that patients with high risk scores had higher proportions of neutrophils and mast resting cell phenotypes. However, immunosuppressive cells, such as follicular helper T cells and CD8 T cells, were elevated in the low-risk group, indicating an immune disability status between the groups.

Immune checkpoints are potential targets for cancer therapy, and inhibitors that block key molecules have shown an impressive efficacy against cancer. In this study, the high-risk group was also correlated with immune checkpoints such as PD-L1, PD-1, CTLA-4, and LAG3. Moreover, we found that some immunosuppressive genes were also elevated in the high-risk group, which further affected immune responses.

However, this signature is associated with some limitations. We analyzed the clinical data base on TCGA and GSE32894 datasets and the ROC values for the hypoxia-related risk signature were not high enough. Further validation of the immune microenvironment and this prognostic signature is required. Other clinical databases and more specific experiments are still needed to verify these findings.

In summary, this is the first study to construct and validate a hypoxia-related risk signature based on 7 HRGs in bladder cancer. This signature reflects the degree of immune microenvironment. This study elucidates on the importance of the hypoxia-related gene signature in estimating the prognosis of cancer patients and may be beneficial in individualized treatment strategies.

## Supplementary Information

**Additional file 1: Figure S1.** Comparisons of 5-year ROC curves for the risk score and other clinical characteristics (A) and individual genes (B).

**Additional file 2: Figure S2.** Kaplan–Meier survival curves for the low- and high-risk groups stratified by clinicopathological variables in the GSE32894 dataset.

**Additional file 3: Figure S3.** Correlation analysis between the hypoxia-related risk signature and immune cell infiltration.

**Additional file 4: Figure S4.** GEPIA survival analysis of ALDOB (A), SDC4 (B), and SLC2A3 (C).

**Additional file 5: Figure S5.** Immunohistochemistry of the 7 HRGs based on the Human Protein Atlas. IHC staining of SLC2A3 (A), ALDOB (C), FOXO3 (E), SDC4 (G), VEGF (I), EGFR (K) and GPC1 (M) in normal tissues. IHC staining of SLC2A3 (B), ALDOB (D), FOXO3 (F), SDC4 (H), VEGF (J), EGFR (L) and GPC1 (N) in tumor tissues.

**Additional file 6: Figure S6.** Immunohistochemical staining shows the expression levels of EGFR and SLC2A3 in bladder cancer tissues and paracancerous tissues. *p < 0.05, **p < 0.01, and ***p < 0.001.

**Additional file 7: Table S1.** Clinical characteristics of bladder cancer patients from TCGA cohort.

**Additional file 8: Table S2.** Clinical characteristics of bladder cancer patients from GSE32894 cohort.

**Additional file 9: Table S3.** Clinical characteristics of 45 bladder cancer patients.

**Additional file 10: Table S4.** Sequences of primers.

**Additional file 11: Table S5.** The correlation and p value of tumour infiltrating immune cells and risk signature.

## Data Availability

The authors declare that the data supporting the findings of this study are available within the article.

## References

[1] Choudhry H, Harris AL (2018). Advances in hypoxia-inducible factor biology. Cell Metab.

[2] Wilson WR, Hay MP (2011). Targeting hypoxia in cancer therapy. Nat Rev Cancer.

[3] Jing X, Yang F, Shao C, Wei K, Xie M, Shen H (2019). Role of hypoxia in cancer therapy by regulating the tumor microenvironment. Mol Cancer.

[4] Bray F, Ferlay J, Soerjomataram I, Siegel RL, Torre LA, Jemal A (2018). Global cancer statistics 2018: GLOBOCAN estimates of incidence and mortality worldwide for 36 cancers in 185 countries. CA Cancer J Clin.

[5] Kamat AM, Hahn NM, Efstathiou JA, Lerner SP, Malmstrom PU, Choi W (2016). Bladder cancer. Lancet.

[6] Theodoropoulos VE, Lazaris A, Sofras F, Gerzelis I, Tsoukala V, Ghikonti I (2004). Hypoxia-inducible factor 1 alpha expression correlates with angiogenesis and unfavorable prognosis in bladder cancer. Eur Urol.

[7] Lu M, Ge Q, Wang G, Luo Y, Wang X, Jiang W (2018). CIRBP is a novel oncogene in human bladder cancer inducing expression of HIF-1alpha. Cell Death Dis.

[8] Xue M, Chen W, Xiang A, Wang R, Chen H, Pan J (2017). Hypoxic exosomes facilitate bladder tumor growth and development through transferring long non-coding RNA-UCA1. Mol Cancer.

[9] Su Y, Yang W, Jiang N, Shi J, Chen L, Zhong G (2019). Hypoxia-elevated circELP3 contributes to bladder cancer progression and cisplatin resistance. Int J Biol Sci.

[10] Terry S, Buart S, Chouaib S (2017). Hypoxic stress-induced tumor and immune plasticity, suppression, and impact on tumor heterogeneity. Front Immunol.

[11] Jubb AM, Buffa FM, Harris AL (2010). Assessment of tumour hypoxia for prediction of response to therapy and cancer prognosis. J Cell Mol Med.

[12] Lee CT, Mace T, Repasky EA (2010). Hypoxia-driven immunosuppression: a new reason to use thermal therapy in the treatment of cancer?. Int J Hyperthermia.

[13] Noman MZ, Desantis G, Janji B, Hasmim M, Karray S, Dessen P (2014). PD-L1 is a novel direct target of HIF-1alpha, and its blockade under hypoxia enhanced MDSC-mediated T cell activation. J Exp Med.

[14] Cubillos-Zapata C, Avendano-Ortiz J, Hernandez-Jimenez E, Toledano V, Casas-Martin J, Varela-Serrano A, et al. Hypoxia-induced PD-L1/PD-1 crosstalk impairs T-cell function in sleep apnoea. Eur Respir J. 2017;50(4). 10.1183/13993003.00833-2017 29051270

[15] Ho PC, Bihuniak JD, Macintyre AN, Staron M, Liu X, Amezquita R (2015). Phosphoenolpyruvate is a metabolic checkpoint of anti-tumor T cell responses. Cell.

[16] Albadari N, Deng S, Li W (2019). The transcriptional factors HIF-1 and HIF-2 and their novel inhibitors in cancer therapy. Expert Opin Drug Discov.

[17] Antoni S, Ferlay J, Soerjomataram I, Znaor A, Jemal A, Bray F (2017). Bladder cancer incidence and mortality: a global overview and recent trends. Eur Urol.

[18] Butt SU, Malik L (2018). Role of immunotherapy in bladder cancer: past, present and future. Cancer Chemother Pharmacol.

[19] Lin W, Wu S, Chen X, Ye Y, Weng Y, Pan Y (2020). Characterization of hypoxia signature to evaluate the tumor immune microenvironment and predict prognosis in glioma groups. Front Oncol.

[20] Zhang Q, Huang R, Hu H, Yu L, Tang Q, Tao Y (2020). Integrative analysis of hypoxia-associated signature in pan-cancer. iScience.

[21] Chai YJ, Yi JW, Oh SW, Kim YA, Yi KH, Kim JH (2017). Upregulation of SLC2 (GLUT) family genes is related to poor survival outcomes in papillary thyroid carcinoma: analysis of data from The Cancer Genome Atlas. Surgery.

[22] Kim E, Jung S, Park WS, Lee JH, Shin R, Heo SC (2019). Upregulation of SLC2A3 gene and prognosis in colorectal carcinoma: analysis of TCGA data. BMC Cancer.

[23] Bu P, Chen KY, Xiang K, Johnson C, Crown SB, Rakhilin N (2018). Aldolase B-mediated fructose metabolism drives metabolic reprogramming of colon cancer liver metastasis. Cell Metab.

[24] Salcher S, Spoden G, Hagenbuchner J, Fuhrer S, Kaserer T, Tollinger M (2020). A drug library screen identifies carbenoxolone as novel FOXO inhibitor that overcomes FOXO_3_-mediated chemoprotection in high-stage neuroblastoma. Oncogene.

[25] Chen LL, Gao GX, Shen FX, Chen X, Gong XH, Wu WJ (2018). SDC4 gene silencing favors human papillary thyroid carcinoma cell apoptosis and inhibits epithelial mesenchymal transition via wnt/beta-catenin pathway. Mol Cells.

[26] Liang H, Xiao J, Zhou Z, Wu J, Ge F, Li Z (2018). Hypoxia induces miR-153 through the IRE1alpha-XBP1 pathway to fine tune the HIF1alpha/VEGFA axis in breast cancer angiogenesis. Oncogene.

[27] Rebouissou S, Bernard-Pierrot I, de Reynies A, Lepage ML, Krucker C, Chapeaublanc E (2014). EGFR as a potential therapeutic target for a subset of muscle-invasive bladder cancers presenting a basal-like phenotype. Sci Transl Med.

[28] Hara H, Takahashi T, Serada S, Fujimoto M, Ohkawara T, Nakatsuka R (2016). Overexpression of glypican-1 implicates poor prognosis and their chemoresistance in oesophageal squamous cell carcinoma. Br J Cancer.

[29] Petrova V, Annicchiarico-Petruzzelli M, Melino G, Amelio I (2018). The hypoxic tumour microenvironment. Oncogenesis.

[30] Damgaci S, Ibrahim-Hashim A, Enriquez-Navas PM, Pilon-Thomas S, Guvenis A, Gillies RJ (2018). Hypoxia and acidosis: immune suppressors and therapeutic targets. Immunology.

[31] Labiano S, Palazon A, Melero I (2015). Immune response regulation in the tumor microenvironment by hypoxia. Semin Oncol.

[32] Facciabene A, Peng X, Hagemann IS, Balint K, Barchetti A, Wang LP (2011). Tumour hypoxia promotes tolerance and angiogenesis via CCL28 and T(reg) cells. Nature.

