# Supplementary figures and images for: Identification and validation of a hypoxia-related prognostic and immune microenvironment signature in bladder cancer
Source: Cancer Cell Int. 2021 May 7;21:251. doi: 10.1186/s12935-021-01954-4 (PMC8103571; doi:10.1186/s12935-021-01954-4)

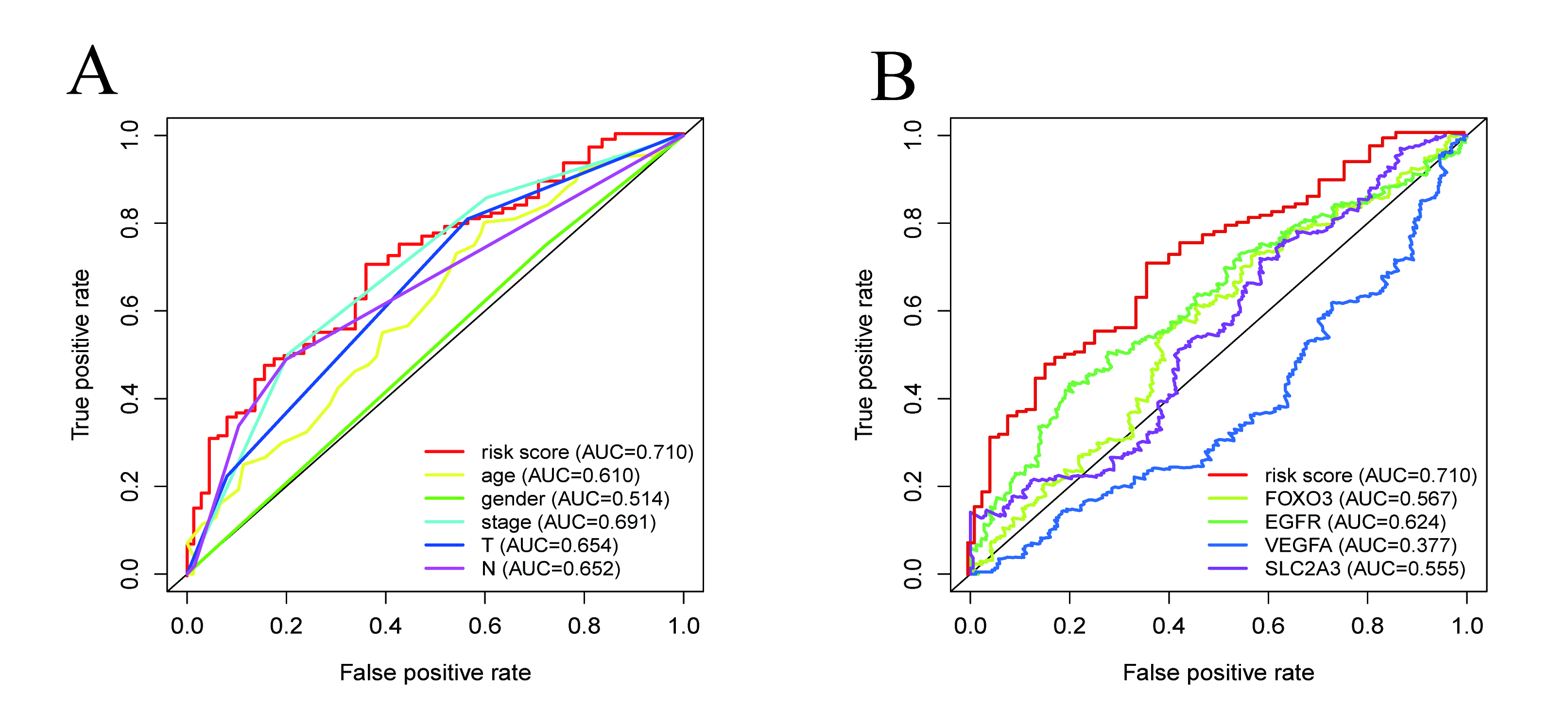

Supplement: Supplementary file 1 — Additional file 1: Figure S1. Comparisons of 5-year ROC curves for the risk score and other clinical characteristics (A) and individual genes (B). [file 12935_2021_1954_MOESM1_ESM.tif]

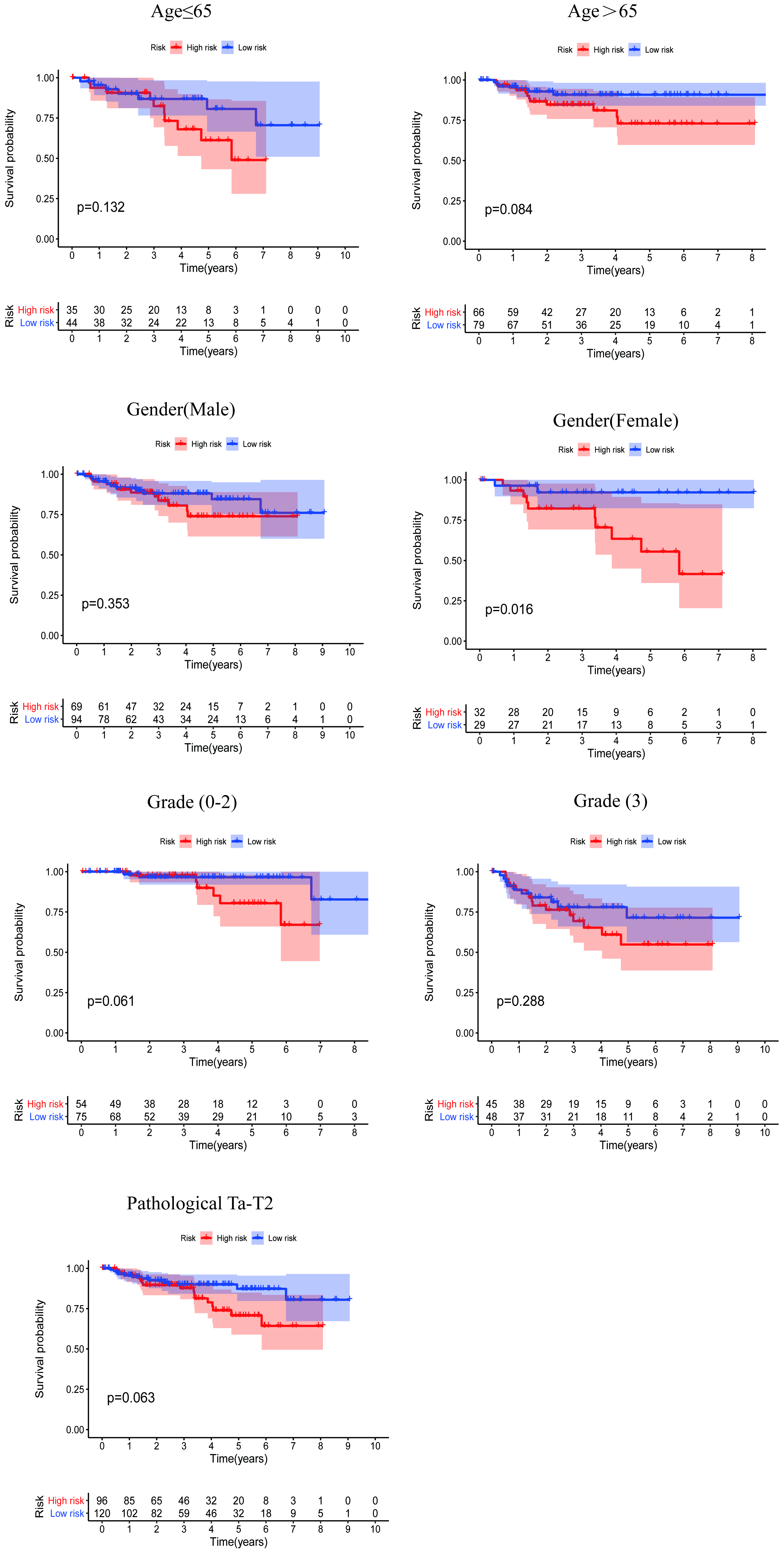

Supplement: Supplementary file 2 — Additional file 2: Figure S2. Kaplan–Meier survival curves for the low- and high-risk groups stratified by clinicopathological variables in the GSE32894 dataset. [file 12935_2021_1954_MOESM2_ESM.tif]

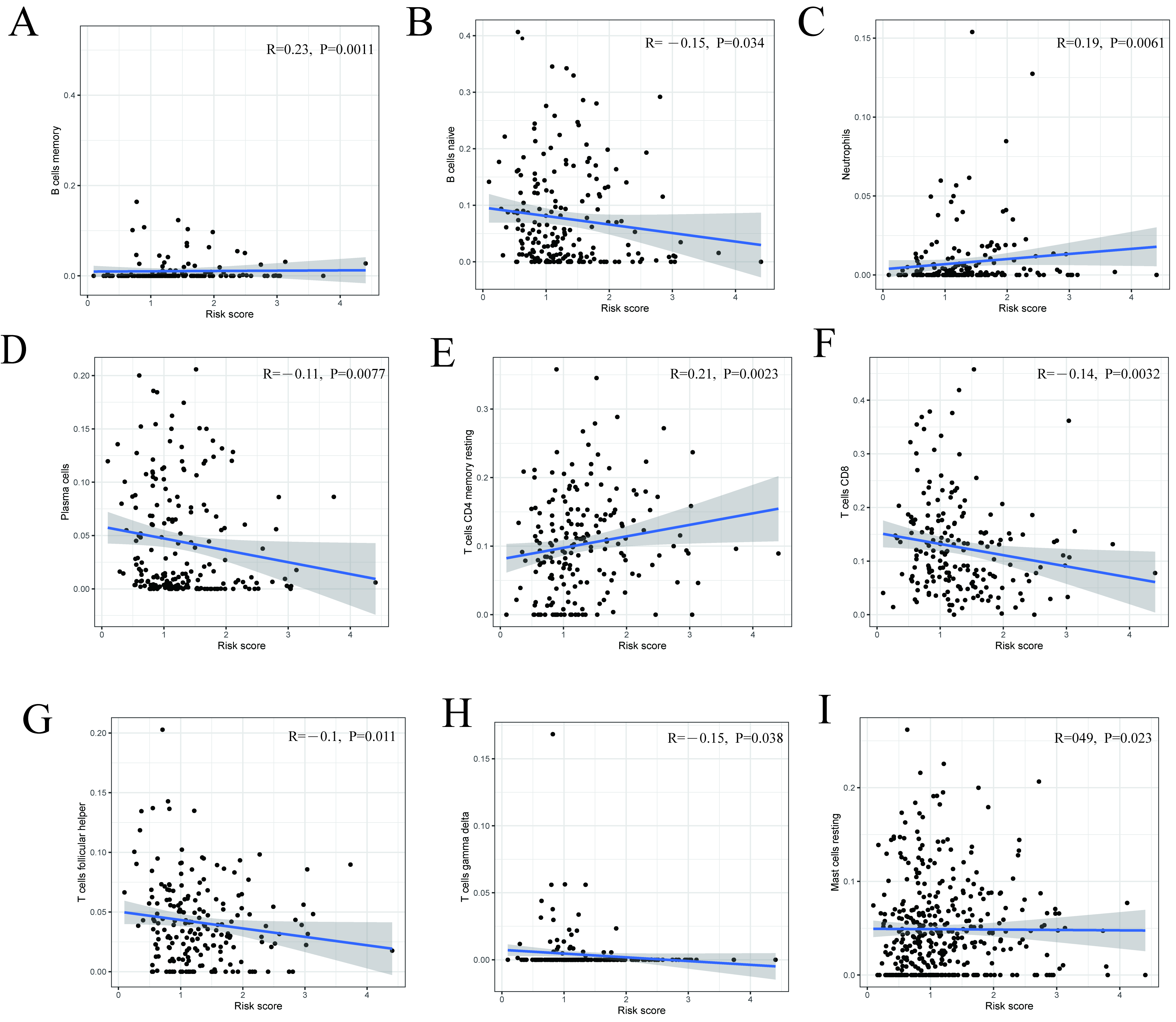

Supplement: Supplementary file 3 — Additional file 3: Figure S3. Correlation analysis between the hypoxia-related risk signature and immune cell infiltration. [file 12935_2021_1954_MOESM3_ESM.tif]

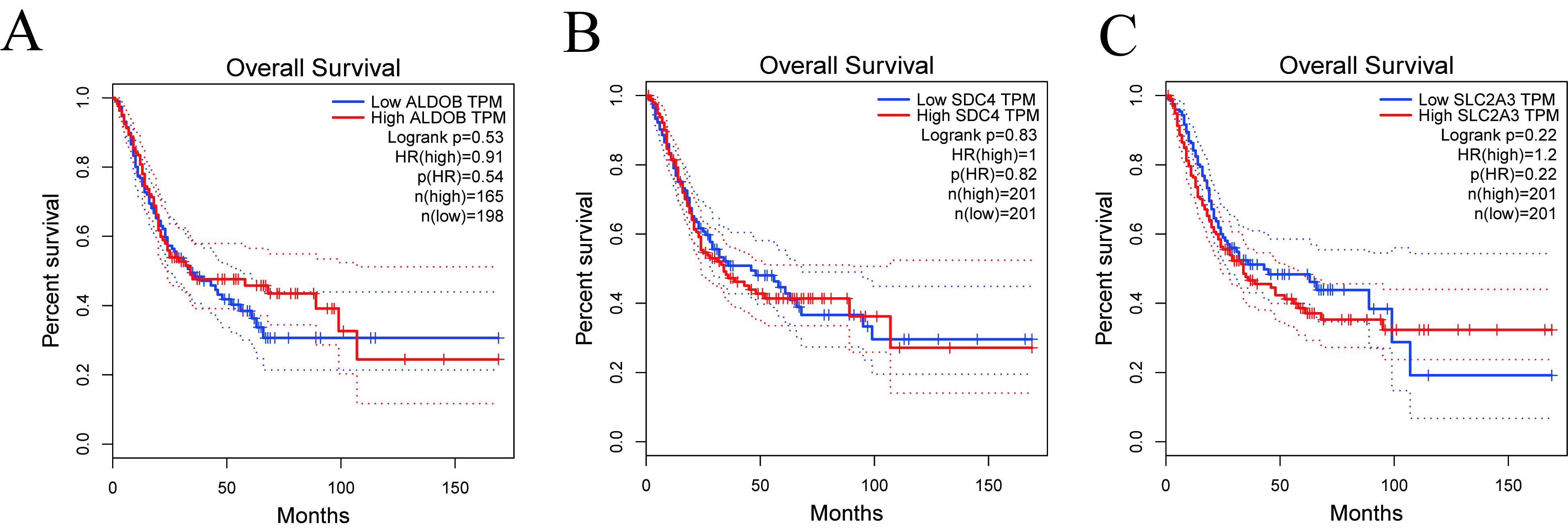

Supplement: Supplementary file 4 — Additional file 4: Figure S4. GEPIA survival analysis of ALDOB (A), SDC4 (B), and SLC2A3 (C). [file 12935_2021_1954_MOESM4_ESM.tif]

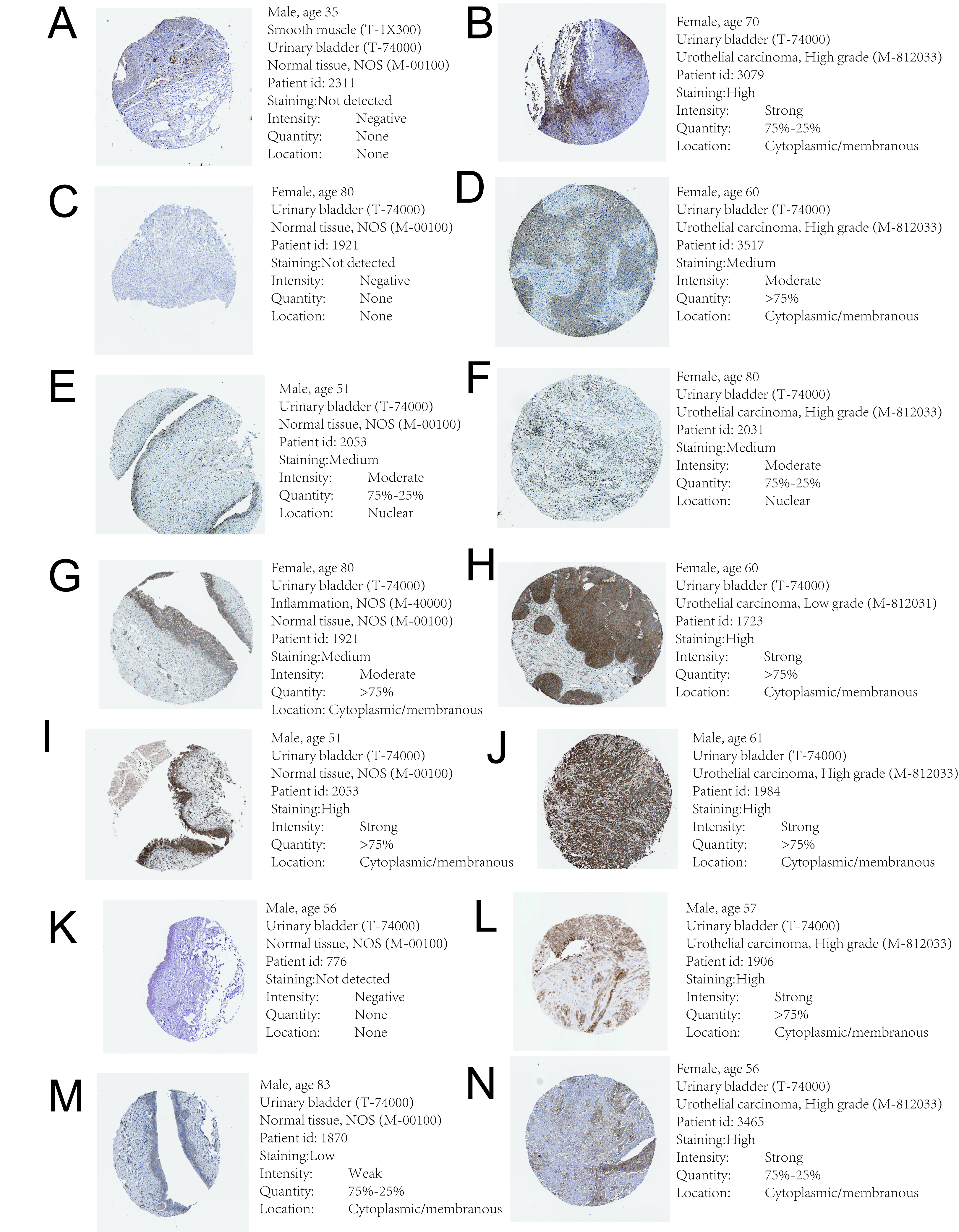

Supplement: Supplementary file 5 — Additional file 5: Figure S5. Immunohistochemistry of the 7 HRGs based on the Human Protein Atlas. IHC staining of SLC2A3 (A), ALDOB (C), FOXO3 (E), SDC4 (G), VEGF (I), EGFR (K) and GPC1 (M) in normal tissues. IHC staining of SLC2A3 (B), ALDOB (D), FOXO3 (F), SDC4 (H), VEGF (J), EGFR (L) and GPC1 (N) in tumor tissues. [file 12935_2021_1954_MOESM5_ESM.tif]

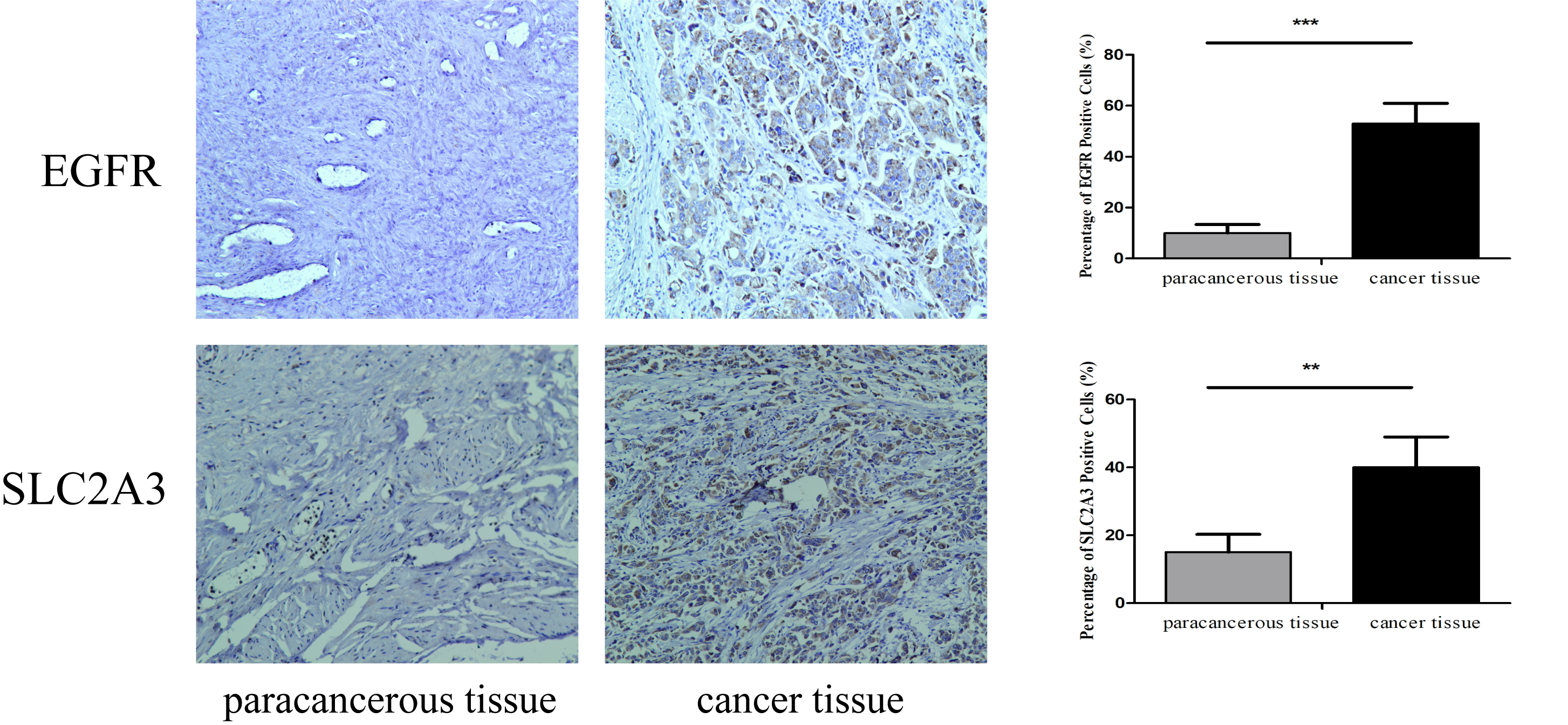

Supplement: Supplementary file 6 — Additional file 6: Figure S6. Immunohistochemical staining shows the expression levels of EGFR and SLC2A3 in bladder cancer tissues and paracancerous tissues. *p < 0.05, **p < 0.01, and ***p < 0.001. [file 12935_2021_1954_MOESM6_ESM.tif]
