# Supplementary material for: Identification and validation of a hypoxia-related prognostic and immune microenvironment signature in bladder cancer
Source: Cancer Cell Int. 2021 May 7;21:251. doi: 10.1186/s12935-021-01954-4 (PMC8103571; doi:10.1186/s12935-021-01954-4)
Supplement: Supplementary file 7 — Additional file 7: Table S1. Clinical characteristics of bladder cancer patients from TCGA cohort. [file 12935_2021_1954_MOESM7_ESM.docx]

**Table S1. Clinical characteristics of bladder cancer patients from TCGA cohort.**

| Clinical characteristics | TCGA cohort |
| --- | --- |
| Age at diagnosis (year)  ≤ 65  ＞ 65  Gender  Male  Female  Grade  Low grade  High grade  T stage  T0-1  T2  T3  T4  N stage  N0  N1  N2  N3  M stage  M0  M1  Stage  Ⅰ  Ⅱ  Ⅲ  Ⅳ | 162  250  304  108  21  388  4  120  196  60  239  47  76  8  196  11  2  131  141  136 |
