# Supplementary material for: Identification and validation of a hypoxia-related prognostic and immune microenvironment signature in bladder cancer
Source: Cancer Cell Int. 2021 May 7;21:251. doi: 10.1186/s12935-021-01954-4 (PMC8103571; doi:10.1186/s12935-021-01954-4)
Supplement: Supplementary file 8 — Additional file 8: Table S2. Clinical characteristics of bladder cancer patients from GSE32894 cohort. [file 12935_2021_1954_MOESM8_ESM.docx]

**Table S2. Clinical characteristics of bladder cancer patients from GSE32894 cohort.**

| Clinical characteristics | GSE32894 cohort |
| --- | --- |
| Age at diagnosis (year)  ≤ 65  ＞ 65  Gender  Male  Female  Grade  G1  G2  G3  T stage  Ta  T1  T2  T3  T4 | 79  145  163  61  45  84  93  110  63  43  7  1 |
