# Supplementary material for: Identification and validation of a hypoxia-related prognostic and immune microenvironment signature in bladder cancer
Source: Cancer Cell Int. 2021 May 7;21:251. doi: 10.1186/s12935-021-01954-4 (PMC8103571; doi:10.1186/s12935-021-01954-4)
Supplement: Supplementary file 9 — Additional file 9: Table S3. Clinical characteristics of 45 bladder cancer patients. [file 12935_2021_1954_MOESM9_ESM.docx]

**Table S3. Clinical characteristics of 45 bladder cancer patients.**

| Clinical characteristics | 45 bladder cancer patients |
| --- | --- |
| Age  ≤ 65  ＞ 65  Gender  Male  Female  Grade  Low grade  High grade  T stage  T1  T2  T3  T4  N stage  N0  N1  N2 | 15  30  36  9  24  21  9  18  13  5  36  7  2 |
