# Supplementary material for: Identification and validation of a hypoxia-related prognostic and immune microenvironment signature in bladder cancer
Source: Cancer Cell Int. 2021 May 7;21:251. doi: 10.1186/s12935-021-01954-4 (PMC8103571; doi:10.1186/s12935-021-01954-4)
Supplement: Supplementary file 10 — Additional file 10: Table S4. Sequences of primers. [file 12935_2021_1954_MOESM10_ESM.docx]

**Table S4**

Primer sequence.

| **Gene Name** | **Primer Sequence** |
| --- | --- |
| **GAPDH** | F: AATGGGCAGCCGTTAGGAAA  R: GCCCAATACGACCAAATCAGAG |
| **SLC2A3** | F: TGGAGAAAACTTGCTGCTGAGA  R: TCAGAGCTGGGGTGACCTTCT |
| **ALDOB** | F: TTCCACGAGACCCTCTACCA  R: GCACCTCCTTGGTCTAACTTG |
| **FOXO3** | F: TGGATGCTGATGGGTTGGAT  R: GTGTCAGTTTGAGGGTCTGC |
| **SDC4** | F: GATGACTTTGAGCTGTCTGGC  R: TATGGTTATCTAGAGGCACCAAGG |
| **VEGFA** | F: ATAAGTCCTGGAGCGTTCCCT  R: TTTAACTCAAGCTGCCTCGC |
| **EGFR** | F: GCATTGATAGAAATGGGCTGC  R: AGGAGCAGGACTGTTTCCAG |
| **GPC1** | F: TGGTGGCTGCTATGTGCG  R: CAGGTGCTCACCCGAGA |

F: Forward; R: Reverse.
