# Supplementary material for: Identification and validation of a hypoxia-related prognostic and immune microenvironment signature in bladder cancer
Source: Cancer Cell Int. 2021 May 7;21:251. doi: 10.1186/s12935-021-01954-4 (PMC8103571; doi:10.1186/s12935-021-01954-4)
Supplement: Supplementary file 11 — Additional file 11: Table S5. The correlation and p value of tumour infiltrating immune cells and risk signature. [file 12935_2021_1954_MOESM11_ESM.docx]

**Table S5. The correlation and p value of tumour infiltrating immune cells and risk signature**

| Symbol | Type | Correlation | P Value |
| --- | --- | --- | --- |
| B cells naive | CIBERSORT | -0.15 | 0.034 |
| B cells memory | CIBERSORT | 0.23 | 0.0011 |
| Plasma cells | CIBERSORT | -0.11 | 0.0077 |
| T cells CD8 | CIBERSORT | -0.14 | 0.0032 |
| T cells CD4 naive | CIBERSORT | -0.11 | 0.031 |
| T cells CD4 memory resting | CIBERSORT | 0.21 | 0.0023 |
| T cells CD4 memory activated | CIBERSORT | 0.086 | 0.11 |
| T cells follicular helper | CIBERSORT | -0.1 | 0.011 |
| T cells regulatory (Tregs) | CIBERSORT | 0.097 | 0.094 |
| T cells gamma delta | CIBERSORT | -0.15 | 0.038 |
| NK cells resting | CIBERSORT | -0.047 | 0.92 |
| NK cells activated | CIBERSORT | 0.019 | 0.86 |
| Monocytes | CIBERSORT | -0.092 | 0.39 |
| Macrophages M0 | CIBERSORT | 0.17 | 0.00095 |
| Macrophages M1 | CIBERSORT | 0.098 | 0.06 |
| Macrophages M2 | CIBERSORT | 0.094 | 0.072 |
| Dendritic cells resting | CIBERSORT | -0.038 | 0.099 |
| Dendritic cells activated | CIBERSORT | -0.19 | 0.038 |
| Mast cells resting | CIBERSORT | 0.049 | 0.023 |
| Mast cells activated | CIBERSORT | -0.017 | 0.96 |
| Eosinophils | CIBERSORT | -0.0016 | 0.44 |
| Neutrophils | CIBERSORT | 0.19 | 0.0061 |
